# A Comprehensive Analysis of the Structural Recognition between KCTD Proteins and Cullin 3

**DOI:** 10.3390/ijms25031881

**Published:** 2024-02-04

**Authors:** Nicole Balasco, Luciana Esposito, Giovanni Smaldone, Marco Salvatore, Luigi Vitagliano

**Affiliations:** 1 Institute of Molecular Biology and Pathology, CNR c/o Department Chemistry, Sapienza University of Rome, 00185 Rome, Italy; 2 Institute of Biostructures and Bioimaging, CNR, 80131 Naples, Italy; luciana.esposito@cnr.it; 3 IRCCS SYNLAB SDN, 80143 Naples, Italy; giovanni.smaldone@synlab.it (G.S.); direzionescientifica.irccssdn@synlab.it (M.S.)

**Keywords:** KCTD proteins, protein structure prediction, oligomeric state, protein structure-function, Cul3 recognition

## Abstract

KCTD ((K)potassium Channel Tetramerization Domain-containing) proteins constitute an emerging class of proteins involved in fundamental physio-pathological processes. In these proteins, the BTB domain, which represents the defining element of the family, may have the dual role of promoting oligomerization and favoring functionally important partnerships with different interactors. Here, by exploiting the potential of recently developed methodologies for protein structure prediction, we report a comprehensive analysis of the interactions of all KCTD proteins with their most common partner Cullin 3 (Cul3). The data here presented demonstrate the impressive ability of this approach to discriminate between KCTDs that interact with Cul3 and those that do not. Indeed, reliable and stable models of the complexes were only obtained for the 15 members of the family that are known to interact with Cul3. The generation of three-dimensional models for all KCTD–Cul3 complexes provides interesting clues on the determinants of the structural basis of this partnership as clear structural differences emerged between KCTDs that bind or do not bind Cul3. Finally, the availability of accurate three-dimensional models for KCTD–Cul3 interactions may be valuable for the ad hoc design and development of compounds targeting specific KCTDs that are involved in several common diseases.

## 1. Introduction

Proteins generally explicate their biological functions through extensive and, frequently, intricate partnerships that may be dictated by subtle energetic effects. Among others, protein–protein interactions certainly deserve a special role in this context as these macromolecules present a remarkable propensity to establish self and hetero associations. In the vast universe of protein folding motifs, several modules specifically dedicated to mediating these interactions have been identified. Among others, the BTB domain (also denoted as POZ or T1), which was originally identified in the *D. melanogaster* Bric-à-Brac, Tramtrack [1], and Broad transcription regulators, deserves a special position as it is widely widespread in most of the metazoan [2,3]. From the structural point of view, this motif is generally characterized by the presence of a single β-sheet surrounded by five α-helices arranged with a β_1_β_2_α_1_α_2_β_3_α_3_α_4_α_5_ topology (Figure S1). Variations of this common motif, consisting of the presence of extra hairpins and helices, have been detected in the different kingdoms of life [2,3]. Moreover, surveys and classifications of BTB domains have also highlighted the ability of this domain to assemble in distinct oligomeric states. Indeed, BTB domains frequently operate as monomers (e.g., Skp1, Elongin C) or dimers (e.g., SPOP, KLHL protein family). Higher oligomeric states have been detected for BTB domains embedded in potassium channels (Kv1.2 family) and in the emerging class of proteins denoted as KCTD ((K)potassium Channel Tetramerization Domain-containing) proteins, which, despite their names, have little involvement in potassium channeling [4,5,6,7,8,9,10,11,12]. The BTBs of the Kv1.2 potassium channels, which are typically denoted as T1, are N-terminal cytosolic domains that assume a tetrameric structure, thus inducing the formation of the tetrameric membrane channel by the rest of the protein [13,14]. In the twenty-five members (KCTD1-21, KCNRG, TNFAIP1, SHKBP1, and BTBD10) of the family, the BTB represents the defining motif of the family being the only structural element present in all members and plays different structural/functional roles [4,5,6]. Although the oligomeric state of all these proteins is yet to be fully characterized, in most of the members the BTB domain promotes self-association that prevalently leads to the formation of pentameric states [7,8,15,16]. The significant sequence/structural similarity of the BTB domains of different KCTDs likely generates heteropentamers whose functional role, although somehow overlooked, seems to be crucial in the regulation of important biochemical/biological processes [17,18,19,20,21]. Moreover, in KCTDs, the articulated roles of these domains go well beyond the self-association and hetero-oligomerization between different BTBs. Indeed, BTB domains mediate important partnerships that play a fundamental role in the diversified functionalities of these proteins. The specific partnerships of the BTB domains of each member of the family virtually dictate its biological function. Most of the BTB domains of the KCTDs tightly bind to Cullin 3 (Cul3) thus making these proteins important adapters in Cullin-RING E3 ubiquitin ligases (CRL) in protein ubiquitination and degradation [4,22,23,24,25,26,27,28,29]. Indeed, some of the KCTD proteins act as adaptors of the E3 ligase system by anchoring with their C-terminal domain the substrate that has to be ubiquitinated and, simultaneously, Cul3 with their BTB domain, thus helping the assembly of the multiprotein complex deputed to the ubiquitin labeling of the substrate. Other members of the family, which are unable to bind Cul3, establish functional partnerships either with the GABA_B2_ receptor thus affecting its signaling [30,31,32], or with transcription factors of the AP-2 family inhibiting their transactivation [33,34,35].

Although, in the last years, important progress has been made, the structural definition of these partnerships is far from being complete. Indeed, how the self-association of the BTB domains in high oligomeric states creates ad hoc, mutually exclusive, hot spots for different partnerships is unknown. Recently, the structural characterizations of the complexes that fragments of the GABA_B2_ receptor form with the BTB domain of KCTD16 [30,31] have provided fundamental clues in the definition of the recognition between KCTD8/KCTD12/KCTD16 and the receptor. On the other hand, no information is available on the binding mode of KCTD1/KCTD15 to AP-2 transcription factors. Finally, insights into the structural basis of the KCTDs–Cul3 recognition have been obtained for very few members of the family by using homology modeling approaches [36,37,38,39]. The global features of KCTD–Cul3 recognition that emerged from these computational studies have been confirmed by the first experimental structure of a KCTD–Cul3 complex, i.e., the KCTD7^BTB^–Cul3, very recently determined by cryo-electron microscopy [40]. Considering the remarkable structural variability of the Cul3-binding KCTD proteins and taking advantage of the recent advent of effective predictive approaches based on machine-learning techniques as implemented in AlphaFold v2.0 (AF) [41,42,43,44,45], we here explored, at the atomic level, the Cul3 recognition of all members of the KCTD family. The validation and quality assessment of the obtained models demonstrate the effectiveness of these predictive approaches and provide a global view of the determinants that allow the functional interaction between KCTDs and Cul3. The analogies and differences in these partnerships can facilitate the specific targeting of KCTD proteins, which are involved in a plethora of severe human diseases [5,6,46,47,48,49,50,51,52,53,54,55,56,57,58,59]. 

## 2. Results

### 2.1. The KCTD–Cul3 Interaction: An Overview of the Available Literature Data

The interaction with Cul3 represents the most common partnership of the BTB domains of KCTDs. To achieve a comprehensive view of the interactions established by the different members of the family we initially surveyed the BioGRID database (https://thebiogrid.org/, accessed on 1 December 2022 and 1 December 2023) (Table 1). The members of the KCTD family that were not reported in this database as Cul3 binders were initially assigned as non-interactors. This preliminary screening was refined by surveying the literature in the field (Table S1). A general agreement was obtained by these two approaches with two significant exceptions. KCTD11 is not reported among Cul3 interactors although this partnership is well characterized [38,39,60]. On the other hand, KCTD12, which was shown to be unable to interact in vitro with Cul3 by isothermal calorimetry [38], is reported among the interactors. In this context, it is important to note that KCTD12 belongs to a cluster of proteins that are involved in the GABA receptor binding and not in the protein ubiquitination [61]. Based on these observations, KCTD11 and KCTD12 were aggregated to the Cul3 interactor and non-interactor ensembles, respectively (Table 1).

It is also worth noting that for some of the KCTD proteins that were not present in the BioGRID database as Cul3-interactors, the inability to bind Cul3 has been directly demonstrated by in vitro experiments. These validated non-interactors are KCTD16 [7,8], KCTD1 [7], and KCTD15 [38].

Most of the interactions reported in the BioGRID database were extracted from large-scale analyses of Cul3 ligands [62,63,64,65,66,67,68,69,70,71]. In addition, direct molecular evidence of the interactions was provided by specific studies for KCTD2 [21,72], KCTD5 [7,8,21,29,37,73], KCTD6 [7,38,74,75], KCTD7 [76,77,78], KCTD10 [8,79,80,81], KCTD13 [82,83], TNFAIP1 [82,84], KCTD17 [8,21,85], and SHKBP1 [8] (Table S1).

Although recent structural studies have shown that KCTD proteins, despite being prevalently pentameric, may assume other oligomeric organizations [8,86], information on the stoichiometry of the KCTD–Cul3 interactions is rather poor. The most important contribution to the field has been provided by Bullock and coworkers who demonstrated that the BTB domains of KCTD5, KCTD10, KCTD13, SHKBP1, and KCTD17 assume a pentameric state when complexed to Cul3 [8].

### 2.2. Prediction of the KCTD7 and Cul3 Complex: Validation of the Approach

Although KCTD proteins have been the subject of several structural characterizations ([7,8,30,31,61,87] and references therein), experimental information about the KCTD–Cul3 interaction has been missing for a long time. Some insightful information on this partnership was obtained from the molecular modeling [36,37,38,39]. The first experimental cryo-electron microscopy (cryo-EM) structure was reported only very recently by Jiang et al. for the complex formed by KCTD7 and Cul3 [40].

To fill in this important gap of information, here we systematically predicted the three-dimensional structures that KCTDs could form with Cul3 using AlphaFold (see methods for details). Since these predictions were made (October–December 2022) before the release of the KCTD7–Cul3 complex (26/7/2023–PDB ID: 8i79) [40], no bias could have been introduced by this experimental structure. Therefore, the comparison of the predicted with the experimental KCTD7–Cul3 structure of the complex may represent an important validation step for the AlphaFold models here presented. Interestingly, the effectiveness of the predictive approach is also suggested by the close similarity of the oligomeric structure of KCTD7 experimentally detected in the KCTD7–Cul3 complex [40] with the predicted pentamer we previously obtained for the unbound KTD7 (https://alphafold.ibb.cnr.it/protein, accessed on 1 December 2023) using the same approach here employed (Figure S3) [86].

#### 2.2.1. AlphaFold KCTD7^BTB^–Cul3 Predicted Structure

**Figure 1:**
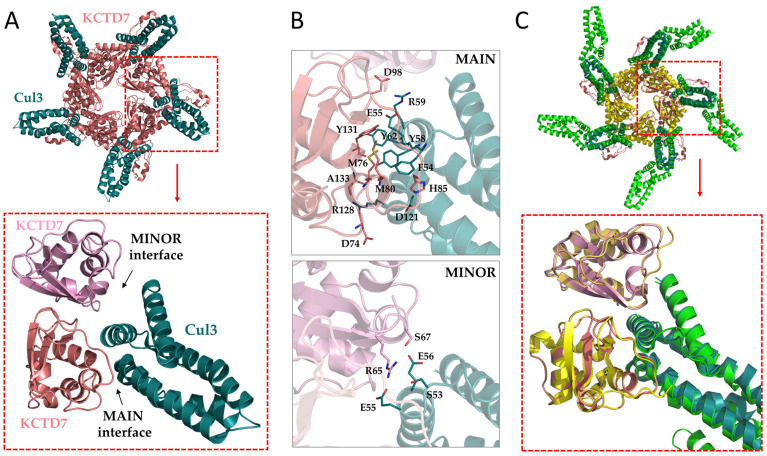
Cartoon representation of the predicted/modeled 5:5 complex between KCTD7^FL^ (pink) and Cul3^17−154^ (dark cyan) (**A**). A snapshot of one of the five equivalent interfaces involving two KCTD chains (main in pink and minor in light pink) and a single Cul3 is shown. Amino acid residues involved in KCTD7–Cul3 interactions at the main and minor interfaces (**B**). Structural superimposition of the experimental (PDB ID: 8i79, KCTD7^FL^ in yellow and Cul3 in green) and predicted (KCTD7^FL^ in pink and Cul3 in dark cyan) KCTD7^FL^–Cul3 complexes (**C**).

Considering that all previous studies indicate that KCTD proteins likely interact with Cul3 only with their BTB domains and the huge amount of atoms of these complexes, we restricted the predictions to the interaction between the BTB domain and the N-terminal region (residues 17-134) of the Cullin (see methods for details). The KCTD7^BTB^–Cul3 5:5 complex predicted by AF presents the expected C5 symmetry (Figure 1A). The analysis of the pLDDT and the PAE matrix (Figure S4), which provides indications about the reliability of the local conformation and the intra/intermolecular distances, strongly suggests that the two proteins form a stable complex. In line with previous suggestions [37], the N-terminal helices (H1–H5) of Cul3 interact with two adjacent BTB subunits within the pentamer (Figure 1A). The analysis of these two interfaces indicates that they are characterized by rather different areas. Indeed, the size of the main and minor interfaces is approximately 720 and 230 Å^2^, respectively. The main interface involves the helix H2 and H5 of Cul3 while only the H2 helix makes contact at the minor interface.

As reported in Table S2 and Figure 1B, the KCTD7–Cul3 partnership is stabilized by different types of polar and apolar interactions. Indeed, H-bonds, which are detected at both the main and minor interfaces, are formed by the side chains of both proteins and the main chain atoms of only KCTD7. Strong electrostatic interactions are established between the side chains of Asp98(KCTD7)–Arg59(Cul3) and His85(KCTD7)–Asp121(Cul3). Close contacts are detected for the hydrophobic moieties of Met76 and Met80 side chains of KCTD7 with Phe54 of Cul3 and of Tyr131 (KCTD7) with Tyr62 (Cul3).

The comparison of the AF model of the complex with the corresponding experimental cryo-electron microscopy structure highlights a remarkable level of similarity both at a global and local level (Figure 1C andFigure S5). Indeed, most of the specific interactions that stabilize the experimental structure of the complex are very well reproduced in the predicted model. The only exceptions are represented by the salt bridges formed by Asp74(KCTD7)–Arg120(Cul3) and Arg84(KCTD7)–Asp121(Cul3) that are present in the cryo-EM complex but not in the AF one.

#### 2.2.2. MD Analysis of the Predicted Model: A Dynamic View of the Interaction

To further investigate the KCTD7–Cul3 recognition mechanism, we performed fully atomistic molecular dynamic (MD) simulations on the AF complex and, for comparative purposes, of the experimental structure (PDB ID: 8i79) [40]. As demonstrated by the inspection of the time evolution of the root mean square deviation (RMSD) values of the trajectory structures compared to the starting model, a rather stable plateau is reached after 20 ns (Figure S6A). The dissection of the MD structures in the different folded domains of the complex (BTB and C-terminal domain CTD of KCTD and Cul3) indicated that minor rearrangements occur for the BTB domain while both Cul3 and the CTD of KCTD7 undergo some significant variations. The limited fluctuation over time of the RMSD values (within 1 Å) observed in the equilibrated region of the trajectories (50–100 ns) indicates that an adequate convergence has been achieved in the MD simulations. It also suggests that globally the complex exhibits limited dynamics. Indeed, no twisting of the CTD vs. the BTB domain of KCTD is observed. This type of motion is instead detectable [87,88] and proposed to be functionally important for KCTD5 [88,89]. The high stability of the complex is also confirmed by the low RMSF values (<2 Å) exhibited by the residues belonging to the structured regions of both KCTD7 and Cul3 (Figure S7). Moreover, the inspection of Figure S7B indicates that the interacting regions (helices H2 and H5 of Cul3) are among those endowed with the lowest flexibility (RMSF values < 1 Å).

**Figure 2:**
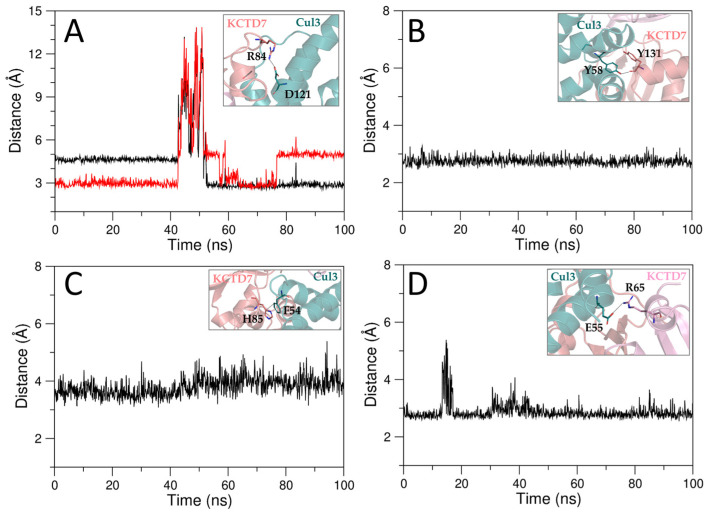
Evolution of the distances between pairs of atoms involved in salt bridge (R84_NH2(red)/NH1(black)-D121_OD2 (**A**)), H-bonding (Y131_O-Y58_OH (**B**)), and hydrophobic (H85_CG-F54_CD2 (**C**)) interactions at the main interface and a salt bridge interaction at the minor interface (R65_NE-E55_OE1 (**D**)) in the MD simulation of the KCTD7^FL^–Cul3 complex (predicted model).

The inspection of the time evolution of the interactions that stabilize the AF-predicted KCTD7–Cul3 interface indicates that they are well preserved throughout the simulation (Figure 2). Furthermore, in the MD simulation of the predicted complex, we observe the formation of the salt bridges involving the side chains of Asp74(KCTD7)–Arg120(Cul3) and Arg84(KCTD7)–Asp121(Cul3) that were present in the experimental structure but missing in the AF model. This observation suggests that the combined use of AF predictions and MD simulations may provide a highly accurate description of protein–protein complexes. Along this line, it is not surprising that an MD simulation carried out using the experimental structure of the complex as a starting model leads to very similar results (Figures S6B and S8).

### 2.3. Large-Scale Prediction of KCTD–Cul3 Interaction

**Figure 3:**
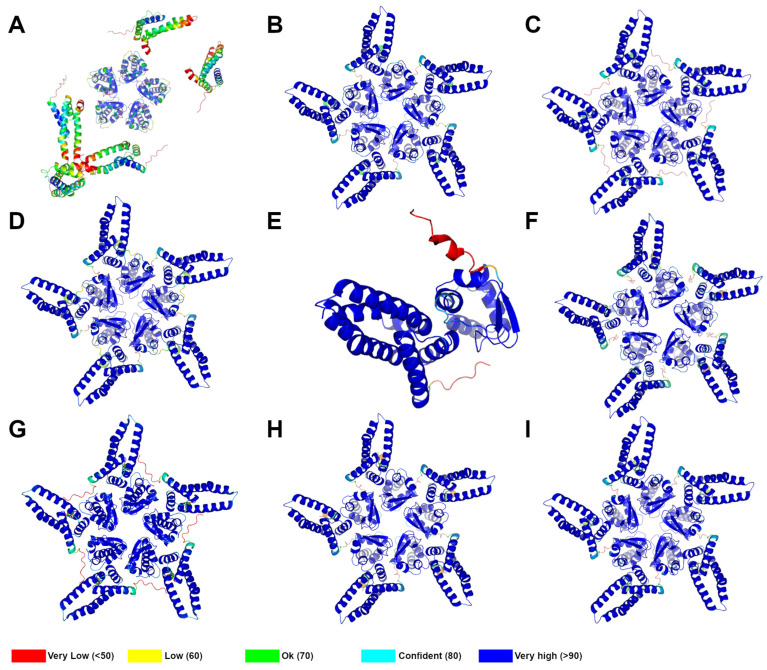
Ribbon representation of AF complexes of selected members of KCTDs (BTB domains) with Cullin3 (residues 17-134). (**A**) KCTD1 (cluster 1B); (**B**) KCTD6 (cluster 2A); (**C**) KCTD21 (cluster 2B); (**D**) KCTD5 (cluster 3); (**E**) BTBD10 (cluster5A); (**F**) KCTD7 (cluster 5B); (**G**) KCTD13 (cluster 6); (**H**) KCTD3 (cluster7); (**I**) KCTD9. Structural models are colored following an AF per-residue confidence metric (pLDDT). A full report of AF-predicted structures for all the members of KCTD clusters that resulted in being bound to Cullin3 is shown in Figure S9.

The excellent agreement observed between the predicted and the experimental structure of the complex formed by KCTD7 and Cul3 prompted us to extend this approach to all members of the family. To evaluate the effectiveness of the approach, the predictions were made for both Cul3-interacting and non-interacting KCTDs (Table 1). As for KCTD7–Cul3, the reliability of the models and the expected stability of the complexes were evaluated by checking the pLDDT values (Figure 3 and Figure S9) and the PAE matrices (Figure S10). Considering the molecular and structural complexity of these proteins and the significant diversification observed in the family, AF predictions and the resulting three-dimensional models are illustrated in the following paragraphs by separately describing each cluster of this protein family (Figure S10). Over the years, different classifications and groupings of KCTDs have been proposed [4,8,61]. Here, the structure-based phylogenetic tree we recently developed, which takes into account previously undetected relationships between protein members, will be used (Figure S2) [61,86].

#### 2.3.1. Cluster 1—KCTD8, KCTD12, KCTD16, KCTD1, and KCTD15

This cluster is composed of two subclusters that embody either KCTD8, KCTD12, and KCTD16 (subcluster 1A) or KCTD1 and KCTD15 (subcluster 1B). Although the members of the two subclusters are involved in different biological processes (see above), their structural characterizations carried out in the last years have highlighted unsuspected structures and sequence similarities also for the CTD [31,86]. Notably, all members of this cluster share the common property of being unable to bind Cul3 (Table 1), although the BTB domains of subcluster 1A bind the GABA_B_ receptor, while those of subcluster 1B bind the AP-2 transcription factors.

The inspection of the PAE matrices of these proteins shows, along with the propensity to form rather stable BTB domains, no tendency of this assembly to interact with Cul3, as proven by the red color (high expected errors) of the region corresponding to the intermolecular BTB–Cul3 interactions (Figure S10). This observation is corroborated by the analysis of the predicted models in which no significant interaction is observed between the BTB domains and the Cul3. A representative model of these meaningless complexes is shown in Figure 3A for KCTD1. These findings are in perfect agreement with the known inability of these proteins to bind Cul3 and suggest that the pentameric association of the BTB of these proteins has specifically evolved to establish other partnerships.

#### 2.3.2. Cluster 2—KCTD6, KCNRG, KCTD11, and KCTD21

Considering structural analogies, the proteins of this cluster have been subdivided into subclusters 2A (KCTD6 and KCNRG) and 2B (KCTD11 and KCTD21). The members of subcluster 2A exhibit different Cul3 binding properties as KCTD6 interacts with Cul3 while KCNRG does not (Table 1). This distinct behavior is fully caught by the AF predictions. Indeed, while the PAE matrices and the pLDDT values corresponding to the AF prediction of the KCTD6^BTB^–Cul3 complex are indicative of a stable pentameric association of the two proteins (Figure 3B and Figure S10), the same predictive indicators suggest the formation of a meaningless complex for KCNRG^BTB^–Cul3 (Figure S10). It is important to note that also the association of the BTB domains of KCNRG leads to a quite loose pentamer. This situation was already detected in the predictions of the ligand-free structure of this protein [86]. In this study, however, the pentameric association of KCNRG was corroborated by MD simulations.

Although the ability to bind Cul3 [51] is correctly predicted for the closely related member of subcluster 2A (KCTD21) (Figure 3C and Figure S10), the relative orientation of KCTD11^BTB^ and the Cullin is significantly different from that detected for the other Cul3-binders KCTDs (see also below). The structural significance of this finding is difficult to assess, considering that the pLDDT values of the Cul3 helices that interact with the BTB are rather poor (Figure S9A) and that even the prediction of the ligand-free full-length KCTD11 was not highly reliable [86]. For some hitherto unknown reasons, the prediction of the KCTD11 structural features is quite difficult for AlphaFold v2.0.

#### 2.3.3. Cluster 3—KCTD2, KCTD5, and KCTD17

The members of this cluster are well-characterized Cul3 interactors. This property is well-predicted by AF, which suggests that all members of the family form stable pentameric complexes with the Cullin (Figure 3D, Figures S9B,C, and S10). It has been recently reported that KCTD5 forms complexes with Cul3 that, although structurally resembling the structure here predicted, are endowed with some intriguing flexibility and asymmetry that may be functionally relevant [89]. No evidence of this asymmetry is evident from AF predictions.

#### 2.3.4. Cluster 4—KCTD4, KCTD19, and KCTD18

Cluster 4 includes the pentameric KCTD4 and the likely monomeric KCTD19, which presents an articulated structural architecture [61] that contains three distinct BTB domains (BTBa, BTBb, and BTBc). Both of them belong to the Cul3 non-interactor group (Table 1). In line with this expectation, AF does not predict the formation of stable complexes of these proteins with the Cullin. Indeed, a meaningless complex is predicted for KCTD4^BTB^ as indicated by the corresponding PAE matrix (Figure S10). Weak possible interactions with Cul3 are predicted for the domains BTBb and BTBc of KCTD19 (Figure S10). However, the juxtaposition of these KCTD19^BTB^–Cul3 structures in the framework of the entire KCTD19 [61] leads to severe steric clashes with the other domains of the protein, thus confirming the lack of any structural significance of these faint complexes.

KCTD18, which is an isolated protein phylogenetically close to cluster 4 [86], is a presumably monomeric protein that interacts with Cul3 (Table 1). In line with this datum, AF predicts a stable 1:1 complex for the KCTD18^BTB^–Cul3 partnership (Figures S9D and S10).

#### 2.3.5. Cluster 5—BTBD10, KCTD20, KCTD7, and KCTD14

This cluster is composed of two rather heterogeneous subclusters, 5A (BTBD10 and KCTD20) and 5B (KCTD7 and KCTD14). While the members of the subcluster 5A are monomeric, those of the cluster 5B are pentameric. Moreover, both BTBD10 and KCTD20 are Cul3 binders whereas only KCTD7 of subcluster 5B interacts with the Cullin (Table 1). Notably, the AF predictions fully adhere to these intricate experimental data. Indeed, stable 1:1 complexes are formed with Cul3 by BTBD10 and KCTD20 (Figure 3E, Figures S9E, and S10). Pentameric 5:5 complexes are formed by KCTD7^BTB^ (Figure 3F, Figures S4 and S10) while a meaningless complex is predicted for the KCTD14^BTB^–Cul3 partnership (Figure S10).

#### 2.3.6. Cluster 6—KCTD10, KCTD13, and TNFAP1

This cluster is formed by proteins that are Cul3 binders. Interestingly, Bullock and coworkers have demonstrated that although the isolated BTB domains of KCTD10 and KCTD13 may assume in the crystalline state unusual tetrameric states, the presence of Cul3 induces the formation of homopentamers for these proteins in a solution [8]. In line with this latter observation, AF predicts stable pentameric 5:5 assemblies for the BTB domains of these proteins in the presence of Cul3 (Figure 3G, Figures S9F,G, and S10).

#### 2.3.7. Non-Canonical KCTDs: Cluster 7—KCTD3, SHKBP1, and KCTD9

This cluster is made of proteins that have been defined as non-canonical KCTDs as they lack any domain presenting a structural resemblance to the CTD domains detected in the other members of the family [61]. On the other hand, in addition to the BTB, they exhibit domains that are not present in other KCTDs [86]. Nevertheless, their BTB domains can bind Cul3 (Table 1). Again, this property is perfectly reproduced by the corresponding AF predictions (Figure 3H,I, Figures S9H, and S10).

### 2.4. Common Traits and Differences in the Cul3 Recognition by KCTDs

**Figure 4:**
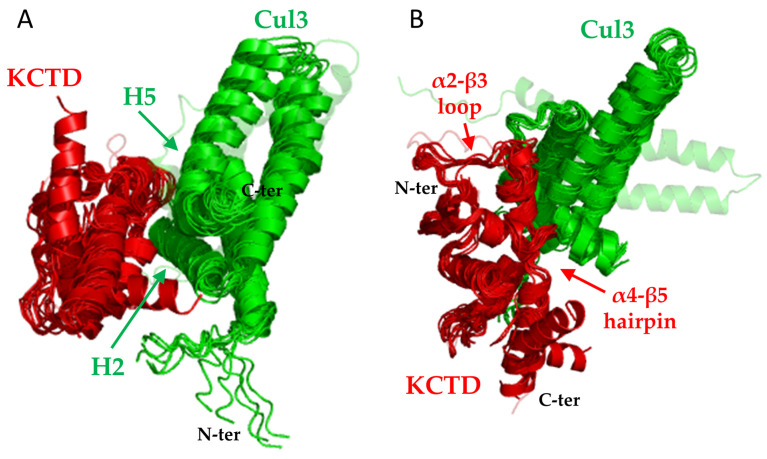
Structural superimpositions of KCTD proteins (red) that are predicted to form stable complexes with Cul3 (green) (see Table 1). Only the main interacting chain of KCTDs is shown. The peculiar binding of Cul3 to KCTD11 is shown in cartoon transparency. Two different views are shown in panels (**A**,**B**).

A comparative analysis of the global features of the KCTD^BTB^–Cul3 complexes indicates a very similar recognition mechanism in 15 out of the 16 Cul3-binding KCTDs, despite the diversity, also in terms of the oligomeric state of the members of this protein family (Figure 4). As anticipated above, the only exception is KCTD11^BTB^ whose modeling for undiscovered reasons is not very reliable. Therefore, this interaction will not be further discussed hereafter. The structural analogy of the complexes is not limited to their overall appearance but also extends to the specific residues of Cul3 involved in the different bindings. As shown in Figure S11, residues of Cul3 involved in H-bonding interactions are largely conserved in the complexes formed. The most conserved of this type of interaction involves residues Phe54, Tyr 58, and Arg59 of the helix 2 (H2) and Asp121 and Arg128 of H5. Residues of Cul3 H1 form H-bonding interactions only sporadically (Figure S11). Cul3 residues are also involved in hydrophobic interactions with the side chains of Phe54, Tyr58, and Tyr 62.

**Figure 5:**
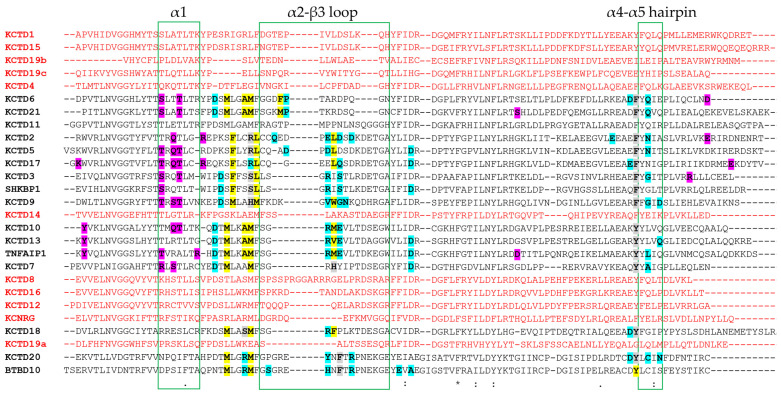
Multiple sequence alignment of the BTB domains of KCTD proteins obtained using CLUSTALW. The symbols (*, :, .) denoting the degree of conservation are reported as provided by CLUSTALW (https://www.genome.jp/tools-bin/clustalw, accessed on 1 December 2023). Residues involved in interactions with Cul3 at the main (cyan for H-bonds, yellow for hydrophobic interactions, grey for both types of contacts) and minor (magenta for H-bonds) interfaces are highlighted for all KCTD^BTB^ forming stable complexes, except for KCTD11^BTB^, whose complex with Cul3 has not been predicted with high reliability (see text). The BTB regions involved in the interaction with Cul3 are indicated with green boxes. KCTD that are not able to form stable complexes with Cul3 are in red.

A rather different situation occurs on the KCTD side. As mentioned above, oligomeric KCTDs anchor the Cullin using two different interfaces (main and minor) involving two distinct subunits of the assembly. The main interface comprises residues of the α2β3 loop and of the α4α5 helical hairpin that anchor the helices H2 and H5 of Cul3 (Figure 4). The minor interface essentially involves residues of the α1 helix and binds helices H1 and H2 of Cul3. In the monomeric KCTDs, only the main interface is present. As shown in Figure 5, while the α1 helix and the α4α5 helical hairpin are relatively conserved in the family, the α2β3 loop is highly variable since significant insertions/deletions are observed. Of these distinct hot spots, most of the interactions are formed by the variable α2β3 loop. Several interactions involving the α1 helix are detected at the minor interface (Figure 5).

## 3. Discussion

KCTD proteins constitute an emerging class of proteins involved in severe and widespread pathologies that include cancer [6], neurological disorders [5], and genetic diseases [20,46,76,90,91,92,93,94,95,96,97,98,99]. The BTB domain located in the N-terminal region of all these proteins constitutes the defining element of the family. As anticipated above, the BTB domain may have in KCTDs the dual role of promoting, in most cases, oligomerization and also cross-oligomerization among different KCTDs and favoring functionally important partnerships with different interactors [16,18,100]. The recent development and release of innovative methodologies that are effective in predicting the three-dimensional structures of proteins and their complexes starting from their sequences are progressively revolutionizing the structural biology [41,42,43]. Indeed, while time-consuming experimental studies of proteins restrict the characterization of individual proteins, predictive methodologies allow the definition of the structural features of entire protein families. By using this approach, we have recently detected previously unknown common features of the C-terminal domains of KCTDs [61]. Moreover, we provided reliable atomic-level models of the oligomeric states of virtually all members of the family (https://alphafold.ibb.cnr.it/, accessed on 1 December 2023) [86]. Here, we further extended this approach to study the KCTD–Cul3 interaction at the global level by predicting the structure with the Cullin for all the members of the family.

The first result of this study is the impressive ability of AlphaFold to discriminate between KCTDs that interact with Cul3 and those that do not. This result is in perfect agreement with the survey here reported of the available experimental data. Indeed, for the 15 members of the family that are known to interact with the Cullin (Table 1), reliable and stable models of the complexes are predicted, independently of the oligomeric state (monomeric or pentameric) of the protein. For the nine KCTDs that do not bind Cul3 or whose binding to the Cullin has not been reported, AF predicts meaningless complexes. Interestingly, stable pentameric complexes have been predicted for the BTB domains of KCTD10, KCTD13, and SHKBP1 that present non-pentameric states in their unbound forms but switch toward the pentameric organization in the presence of Cul3 [8].

**Figure 6:**
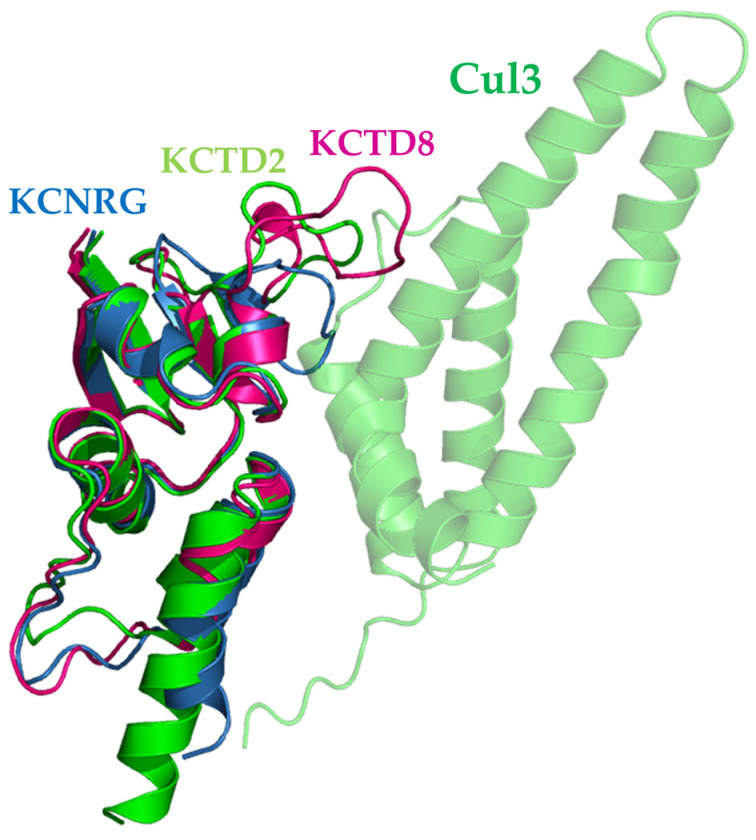
Structural superimpositions of the BTB domains of KCTD8 (magenta) and KCNRG (blue) to KCTD2^BTB^ bound to Cul3 in the predicted complex (green). Only the main interacting chain of KCTD2^BTB^ is shown.

Except for KCTD11, whose complex with Cul3 has not been predicted with high reliability (see above), all KCTD–Cul3 complexes present very similar global and local features. Indeed, most of the crucial, either polar or apolar interactions, involve similar residues on both KCTD and Cul3 sides (Figure 5 and Figure S11). The generation of all three-dimensional models for KCTD^BTB^–Cul3 complexes also provides the opportunity the better define the structural basis of the Cul3 binding in the KCTD family. Although interesting insights on the determinants of Cul3 binding have been obtained based on sequence analysis [40] and/or the inspection of only a few three-dimensional structures generated by homology modeling [37,38], the availability of detailed structural models for all members of the family and for their complexes with Cul3 provides new clues on the intrinsic structural properties of the regions directly involved in the binding and on the sequence/structural basis why some members of the family are unable to bind the Cullin. For KCTD19, the intricate protein architecture prevents the binding of its BTB domains to Cul3 in modes that resemble those detected in other KCTD–Cul3 complexes. Of particular interest is the analysis of the structures adopted by the α2β3 loop in the other KCTDs that do not bind Cul3. In some cases (KCTD8 and KCTD16) the rather long size of this loop prevents the binding of the Cullin (Figure 6 and Figure S12). In other cases (KCTD12, KCNRG, and KCTD14), the presence of secondary structure elements within the loop makes it too rigid for establishing interactions (Figure 6 and Figure S12). Similarly, the rigidity that the presence of a proline residue in the corresponding sequences of KCTD1 and KCTD15 entails may be responsible for the inability of these proteins to bind to Cul3 (Figure S12). Cul3 binders and non-binders present a widespread degree of sequence identities of the BTB domains (Figure S13) and cannot be well discriminated using global sequence similarities. As suggested by the present analysis, local and diversified effects likely dictate the ability/inability to bind the Cullin within the KCTD protein family.

The involvement of the KCTD–Cul3 partnership in many physio-pathological states may suggest that they could represent an interesting target for therapeutical applications as done for the BTB-containing protein BCL6 [101]. Although the BTB domain of KCTDs has been successfully targeted with small peptides [102,103], a lack of specificity of compounds targeting the BTB domain of KCTD proteins can be envisaged due to the similarity of the Cul3 recognition mechanism exploited by these proteins. In this framework, the availability of accurate three-dimensional models for all KCTD–Cul3 interactions may be valuable for the ad hoc design and development of compounds targeting specific KCTDs.

Finally, although the approach applied here likely misses some dynamic aspects as those recently detected for the KCTD5–Cul3 complexes [89], the ability of AF predictions to perfectly discriminate Cul3 binders from non-binders suggests that this approach could be also effective for the prediction of the KCTD^CTD^ partners for which current experimental data are very limited [31,40,89]. Present findings also support the proposed application of this methodology in the proteome and interactome scales [104].

## 4. Methods and Materials

### 4.1. AlphaFold Predictions

Three-dimensional structures of KCTD^BTB^–Cul3 complexes were predicted using the AlphaFold v2.0 algorithm as implemented on the Colab server (https://colab.research.google.com/github/sokrypton/ColabFold/blob/main/AlphaFold2.ipynb, accessed on 1 October 2022) [41]. The definition of the KCTD sequence region corresponding to the BTB domain is taken from the study of Esposito et al. [61], while the interacting N-terminal region (residues 17-134) of Cul3 was considered in the predictions. A longer portion of Cul3 including its first six α-helices (H1–H6, residues 17-154) have been considered for the KCTD7^FL^–Cul3 complex. Predictions were carried out without considering any homologous experimental template (template_mode: none) and with three as the number of recycles and using AlphaFold-multimer v.2. The best-predicted model (rank 1) out of the five computed by AF is considered throughout the present work. The reliability of the AF predictions was assessed by analyzing the Local Distance Difference Test (LDDT) score and the Predicted Aligned Error (PAE) matrices reported for each predicted structure. The model of the complex between the full-length (BTB+hinge+CTD referred to as the full-length FL protein) KCTD7 (residues 50-289) and Cul3 (residues 17-154) was built by superimposing the AF model of the FL [86] protein to the predicted reduced KCTD7^BTB^–Cul3 complex.

### 4.2. Molecular Dynamics Simulations

Fully atomistic MD simulations were performed on the KCTD7^FL^–Cul3 complex using, as starting models, both the predicted/modeled (see above) and the cryo-EM (PDB ID: 8i79) structures. The experimental structure of mouse KCTD7^FL^ was integrated into its missing parts by the AF model [86]. The few mouse-specific amino acid residues present in KCTD7 were replaced with those present in the human sequence. Since Asp26 is the first residue of Cul3 in the experimental structure, the portion 26-154 of Cul3 has been considered in the MD study.

The GROMACS software (version 2022.3) with Amber99sb as an all-atom force field was used [105]. The protein models were solvated with water molecules of the TIP3P model in triclinic boxes and neutralized with counterions (sodium or chloride). The Particle Mesh Ewald (PME) method (0.16 nm grid spacing) was used to treat the electrostatic interactions [106], whereas a cut-off of 10 Å was applied for Lennard–Jones interactions. The LINCS algorithm was used to constrain bond lengths [107]. Systems were energy minimized using the steepest descent for 50,000 steps. Then, they were equilibrated in two steps. The temperature was raised to 300 K in 500 ps (NVT ensemble), and then the pressure was equilibrated at 1 atm in 500 ps (NpT ensemble). The Velocity Rescaling and Parrinello−Rahman algorithms were applied to control temperature and pressure, respectively. For each system, a single MD production run (timescale of 100 ns) was performed at a constant temperature (300 K) and pressure (1 atm) using an integration time step of 2 fs. Structural analyses of MD trajectories were performed using GROMACS tools and the Visual Molecular Dynamics (VMD) program [108]. Figures of structural models were generated using the PyMOL molecular visualization program. Plots were generated using Xmgrace v50125 (https://plasma-gate.weizmann.ac.il/Grace/, accessed on 1 December 2023).

## Figures and Tables

**Table 1:** AF predictions of KCTD^BTB^–Cul3 complexes. Cul3-binders and non-binders, as reported in the biomedical interaction repository BioGRID (see Table S1), are colored in green and red, respectively. A full explanation of the table is reported in the main text (Section 2.1). See Figure S2 for the definition of clusters.

Cluster	Protein Complex	Selected Stoichiometry for the Prediction	AF Prediction
1A	KCTD8^BTB(E44-L145)^–Cul3	5:5	No stable complex detected
	KCTD12^BTB(P33-A131)^–Cul3	5:5	No stable complex detected
	KCTD16^BTB(E25-T123)^–Cul3	5:5	No stable complex detected
1B	KCTD1^BTB(A30-T133)^–Cul3	5:5	No stable complex detected
	KCTD15^BTB(A56-R162)^–Cul3	5:5	No stable complex detected
2A	KCTD6^BTB(D12-D107)^–Cul3	5:5	Stable complex detected
	KCNRG^BTB(E5-Q104)^–Cul3	5:5	No binding observed
2B	KCTD11^BTB(G14-A123)^–Cul3	5:5	Stable complex detected *
	KCTD21^BTB(P4-K108)^–Cul3	5:5	Stable complex detected
3	KCTD2^BTB(R72-T178)^–Cul3	5:5	Stable complex detected
	KCTD5^BTB(V42-T149)^–Cul3	5:5	Stable complex detected
	KCTD17^BTB(G30-V135)^–Cul3	5:5	Stable complex detected
-	KCTD18^BTB(D12-S118)^–Cul3	1:1	Stable complex detected
4	KCTD4^BTB(T33-L135)^–Cul3	5:5	No stable complex detected
	KCTD19^BTBa(D13-E107)^–Cul3	1:1	No stable complex detected
	KCTD19^BTBb(V172-M258)^–Cul3	1:1	No stable complex detected
	KCTD19^BTBc(Q396-Q487)^–Cul3	1:1	No stable complex detected
5A	BTBD10^BTB(M149-C266)^–Cul3	1:1	Stable complex detected
	KCTD20^BTB(E117-C216)^–Cul3	1:1	Stable complex detected
5B	KCTD7^BTB(P50-N143)^–Cul3	5:5	Stable complex detected
	KCTD14^BTB(T33-D124)^–Cul3	5:5	No stable complex detected
6	KCTD10^BTB(Y33-Q129)^–Cul3	5:5	Stable complex detected
	KCTD13^BTB(K41-E142)^–Cul3	5:5	Stable complex detected
	TNFAIP1^BTB(K28-S130)^–Cul3	5:5	Stable complex detected
7	KCTD3^BTB(E18-L115)^–Cul3	5:5	Stable complex detected
	SHKBP1^BTB(E19-R118)^–Cul3	5:5	Stable complex detected
-	KCTD9^BTB(D89-S191)^–Cul3	5:5	Stable complex detected

* As explained in the text, the reliability of this complex is not very high.

## Data Availability

The coordinates of the models described in the present manuscript will be available upon request to the authors and will be submitted to the web server https://alphafold.ibb.cnr.it/.

## Supplementary Materials

The following supporting information can be downloaded at: https://www.mdpi.com/article/10.3390/ijms25031881/s1.
