# Supplementary material for: A Comprehensive Analysis of the Structural Recognition between KCTD Proteins and Cullin 3
Source: Int J Mol Sci. 2024 Feb 4;25(3):1881. doi: 10.3390/ijms25031881 (PMC10856315; doi:10.3390/ijms25031881)
Supplement: Supplementary file 1 [file ijms-25-01881-s001.zip › ijms-2814384-supplementary.pdf]

Supplementary material for:

A comprehensive analysis of the structural recognition between KCTD proteins and Cullin 3

Nicole Balasco [1,*,†], Luciana Esposito [2,†], Giovanni Smaldone [3], Marco Salvatore [3] and Luigi Vitagliano [2,#]

[1]Institute of Molecular Biology and Pathology, CNR c/o Department Chemistry, Sapienza University of Rome, 00185 Rome, Italy
[2]Institute of Biostructures and Bioimaging, CNR, 80131 Naples, Italy
[3]IRCCS SYNLAB SDN, 80143 Naples, Italy
† These authors contributed equally to this work.
* Correspondence: nicole.balasco@cnr.it (N.B.); luigi.vitagliano@cnr.it (L.V.)

Table S1. Detection of KCTD-Cul3 binders from the BioGRID database (https://thebiogrid.org/ accessed on December 2022 and 2023).

Large scale studies	
**Detected protein**	**Reference**
KCTD12, KCTD21, KCTD6, KCTD2, KCTD5, KCTD17, KCTD18, BTBD10, KCTD20, KCTD7, KCTD10, KCTD13, TNFAIP1, KCTD3, SHKBP1, KCTD9	Bennett et al. [1]
KCTD6, KCTD2, BTBD10, KCTD20, KCTD10, KCTD13, TNFAIP1, KCTD3, KCTD9	Huttlin et al. [2]
KCTD6, KCTD2, KCTD5, KCTD17, KCTD18, BTBD10, KCTD20, KCTD10, KCTD13, TNFAIP1, KCTD3, SHKBP1, KCTD9	Kouranti et al. [3]
KCTD6, KCTD17, BTBD10, KCTD20, KCTD10, KCTD13, TNFAIP1, KCTD3, KCTD9	Huttlin et al. [4]
KCTD6, KCTD7, KCTD10, KCTD13, SHKBP1, KCTD9	Luck et al. [5]
KCTD2, KCTD5, KCTD17, BTBD10, KCTD20, KCTD10, KCTD13, KCTD3, KCTD9	Chatrathi et al. [6]
KCTD5, KCTD17, KCTD10, KCTD13, SHKBP1	Pinkas et al. [7]
KCTD6, KCTD5, KCTD17, KCTD9	Ji et al. [8]
KCTD6, KCTD13, TNFAIP1, KCTD9	Rolland et al. [9]
KCTD5, KCTD17, TNFAIP1, KCTD9	Kato et al. [10]
KCTD2. KCTD5, KCTD17	Young et al. [11]
**Specific studies**	
KCTD6	Heride et al. [12]<br>Smaldone et al. [13]<br>Lange et al. [14]
KCTD2	Kim et al. [15]
KCTD5	Balasco et al. [16]<br>Bayón et al. [17]<br>Cho et al. [18]
KCTD17	Kasahara et al. [19]
KCTD7	Wang et al. [20]<br>Staropoli et al. [21]<br>Metz et al. [22]
BTBD10, SHKBP1	Wang et al. [23]
KCTD10	Olma et al. [24]<br>Ren et al. [25]<br>Dubiel et al. [26]<br>Nagai et al. [27]
KCTD13, TNFAIP1	Chen et al. [28]
KCTD13	Ibeawuchi et al. [29]
TNFAIP1	Hu et al. [30]

Table S2. Interactions detected at one of the five equivalent interfaces involving two KCTD chains (chain A as main and chain F as minor) and a single Cul3 (chain E) detected in the predicted KCTD7-Cul3 complex.

KCTD7	Cul3
**Chain A**	**Chain E**
**H-bonding/salt-bridge interactions**	
D74_O	R128_NH2
H85_NE2	D121_OD2
D98_OD1	R59_NH2
Y131_O	Y58_OH
Y131_OH	E55_OE2
A133_O	R128_NE
**Hydrophobic interactions**	
M76, M80, H85	F54
M76, Y131	Y58
Y131	Y62
**Chain F**	**Chain E**
**H-bonding/salt-bridge interactions**	
R65_NH2	E55_OE1
R65_NH2	S53_OG
S67_OG	E56_OE2

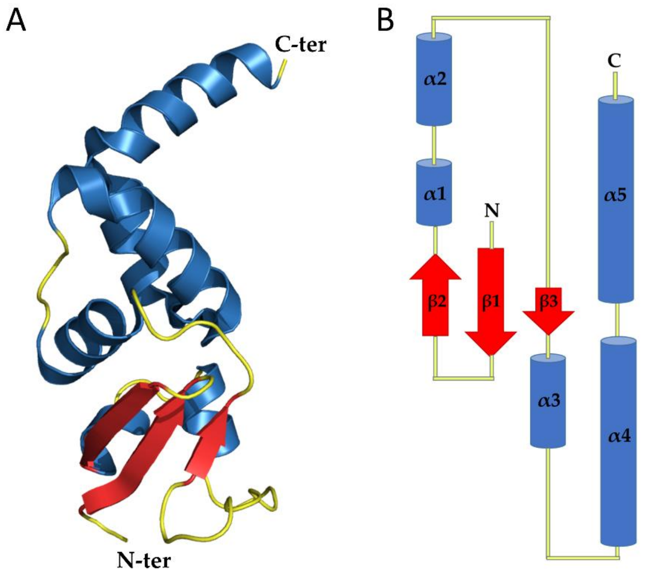

Figure S1. Cartoon representation (A) and topology (B) of the BTB architecture (β1β2α1α2β3α3α4α5). As representative example, the BTB domain of KCTD7 (PDB ID: 8i79) is shown.

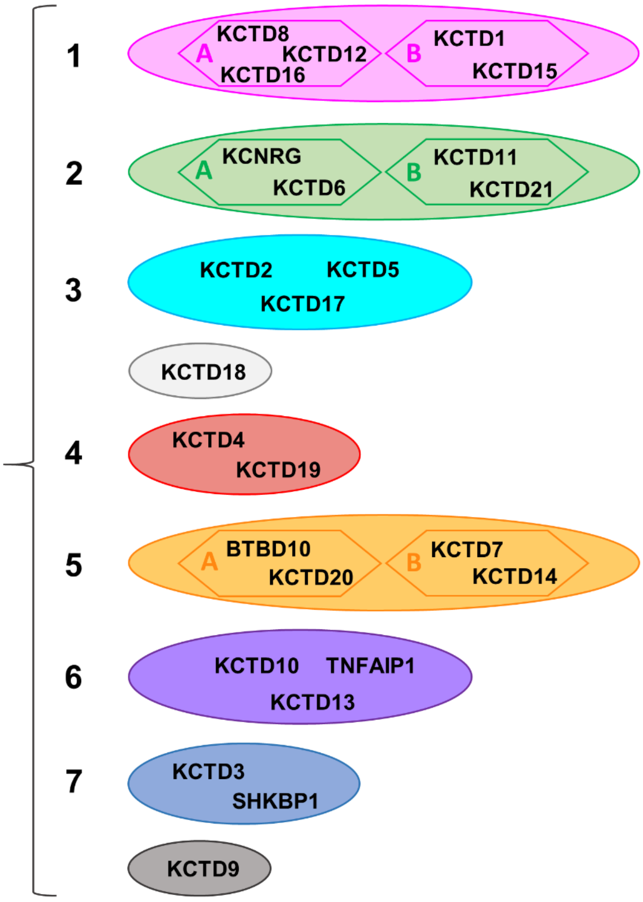

Figure S2. Schematic representation of the structure-based clusters of KCTD proteins derived from Esposito et al. [32,33].

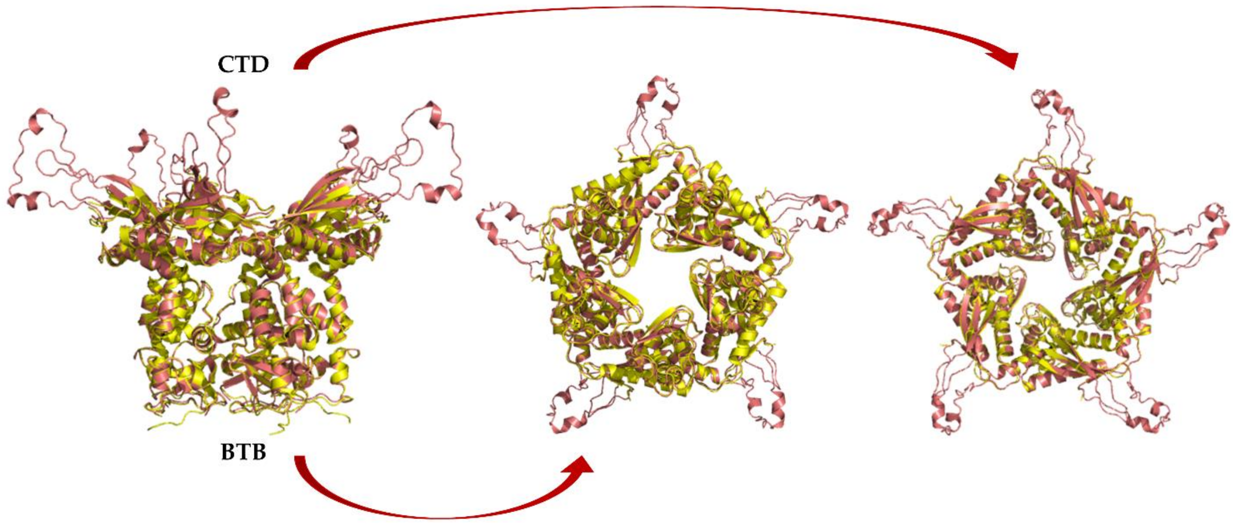

Figure S3. Structural superimposition of the experimental (extracted from the KCTD7-Cul3 complex - PDB ID: 8i79, yellow) [31] and predicted (pink) [32] KCTD7^{FL} structures.

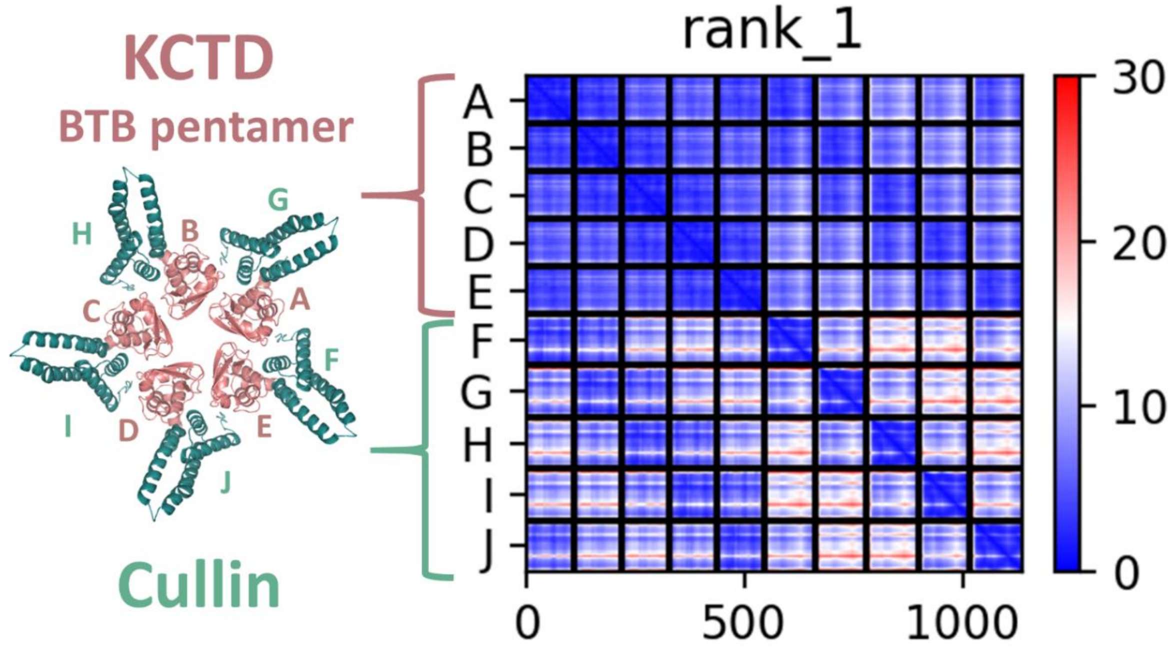

Figure S4. PAE matrix of AF complex between KCTD7 (BTB domain) and Cul3 (residues 17-134). Only the best AF prediction (rank 1) is shown.

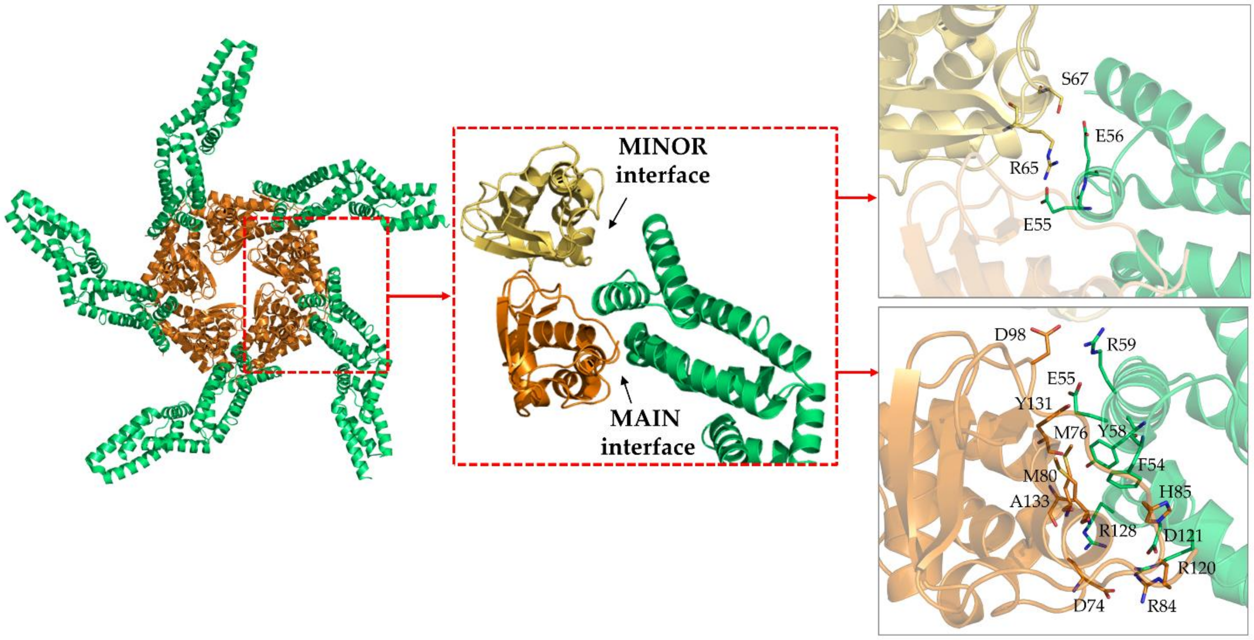

Figure S5. Cartoon representation of the experimental 5:5 complex between KCTD7^{FL} (orange) and Cul3 (green) [31]. A snapshot on one of the five equivalent interfaces involving two KCTD chains (main in orange and minor in yellow) and a single Cul3 is shown and the amino acid residues involved in the interactions at the main/minor interfaces are shown.

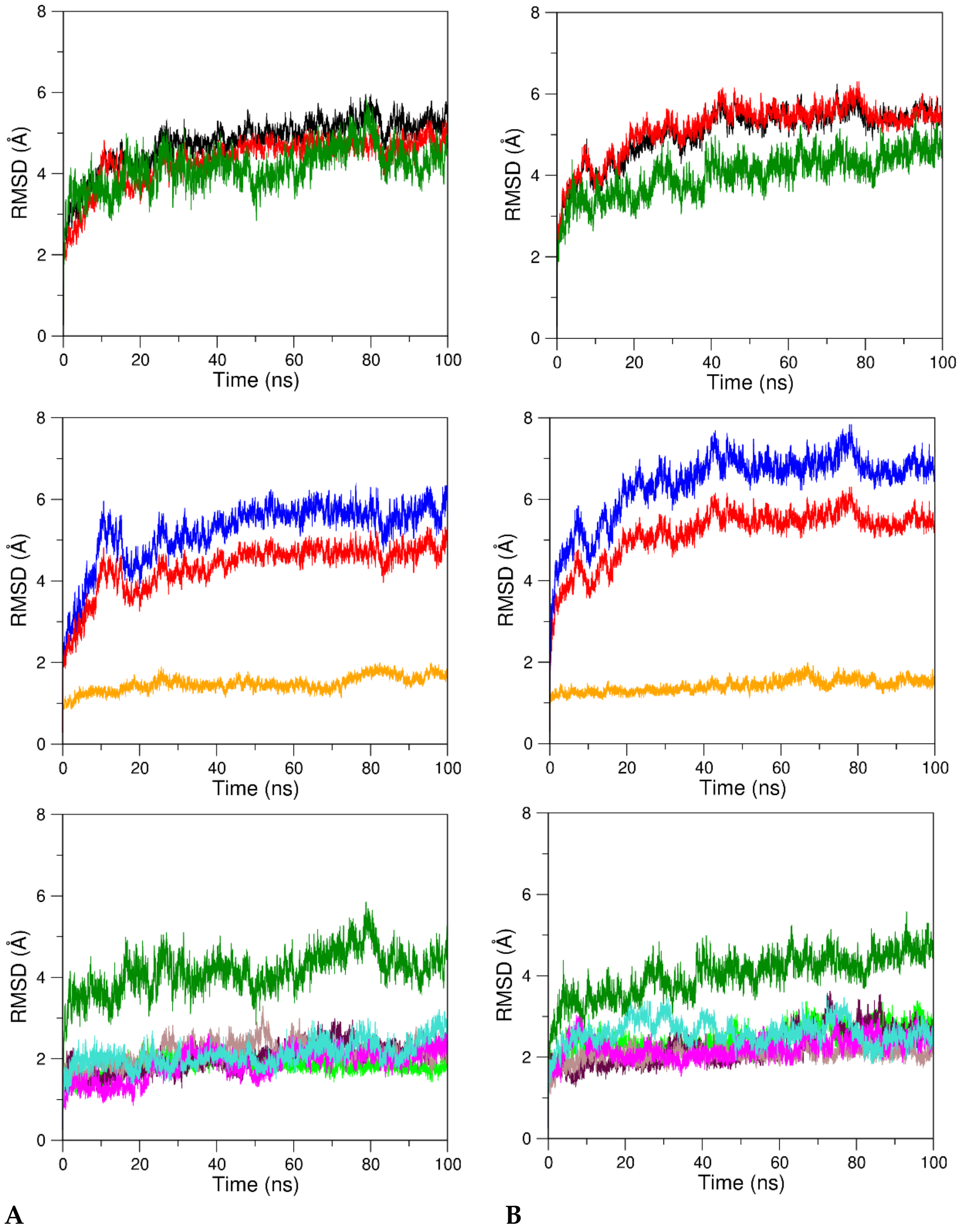

Figure S6. Cα RMSD values of trajectory structures against the starting model calculated on the whole KCTD7^{FL}-Cul3 complex (black), on KCTD7 pentamer (FL in red, BTB in orange and CTD in blue), and Cul3 chains (all chains together in green, the five individual chains in magenta, light green, grey, cyan and violet) in the MD simulations of the predicted (A) and experimental (B, PDB ID: 8i79) KCTD7-Cul3 complex.

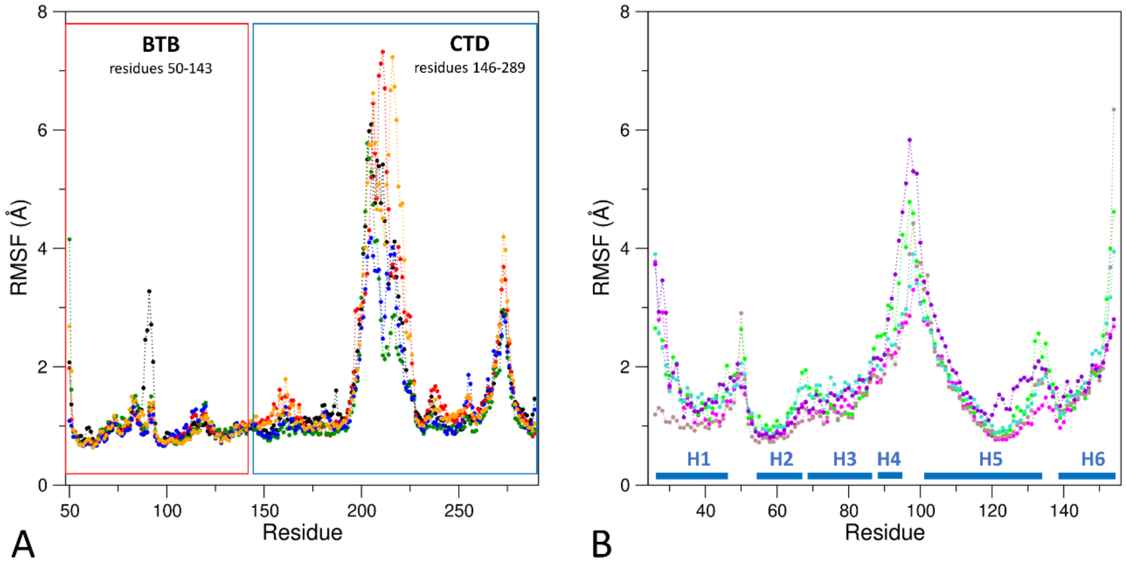

Figure S7. RMSF (Root mean square fluctuation) values computed on the C$^{\alpha}$ atoms of residues of KCTD7 (A) and Cul3 (B) in the equilibrated region (50-100 ns) of the trajectory in the MD simulation of the predicted KCTD7-Cul3 complex.

.

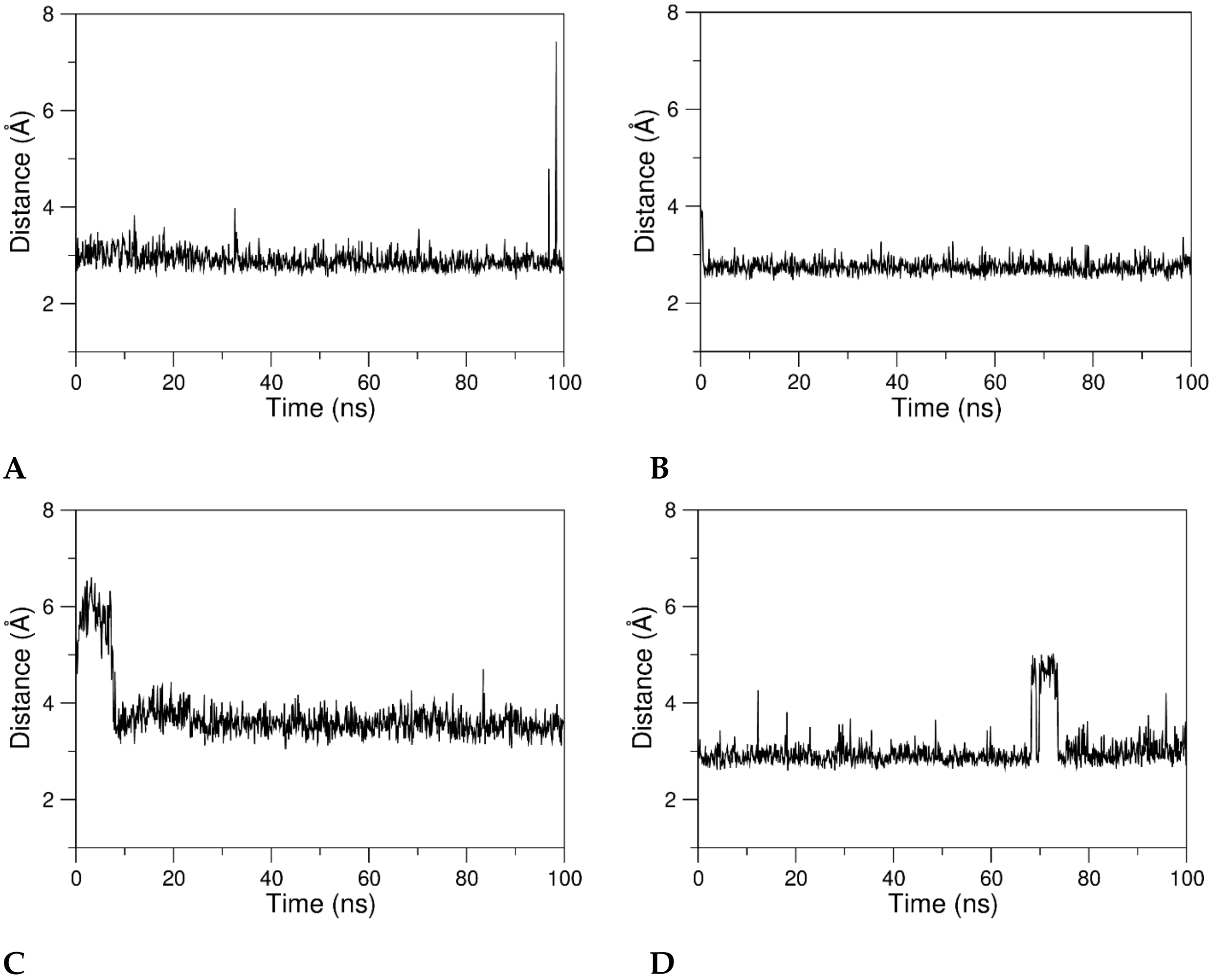

Figure S8. Evolution of the distances between pairs of KCTD7-Cul3 atoms involved in salt bridge (R84_NH2-D121_OD2 (A)), H-bonding (Y131_O-Y58_OH (B)), and hydrophobic (H85_CG-F54_CD2 (C)) interactions at the main interface and a salt bridge interaction at the minor interface (R65_NE-E55_OE1 (D)) in the MD simulation of the experimental KCTD7^{FL}-Cul3 complex (PDB ID: 8i79).

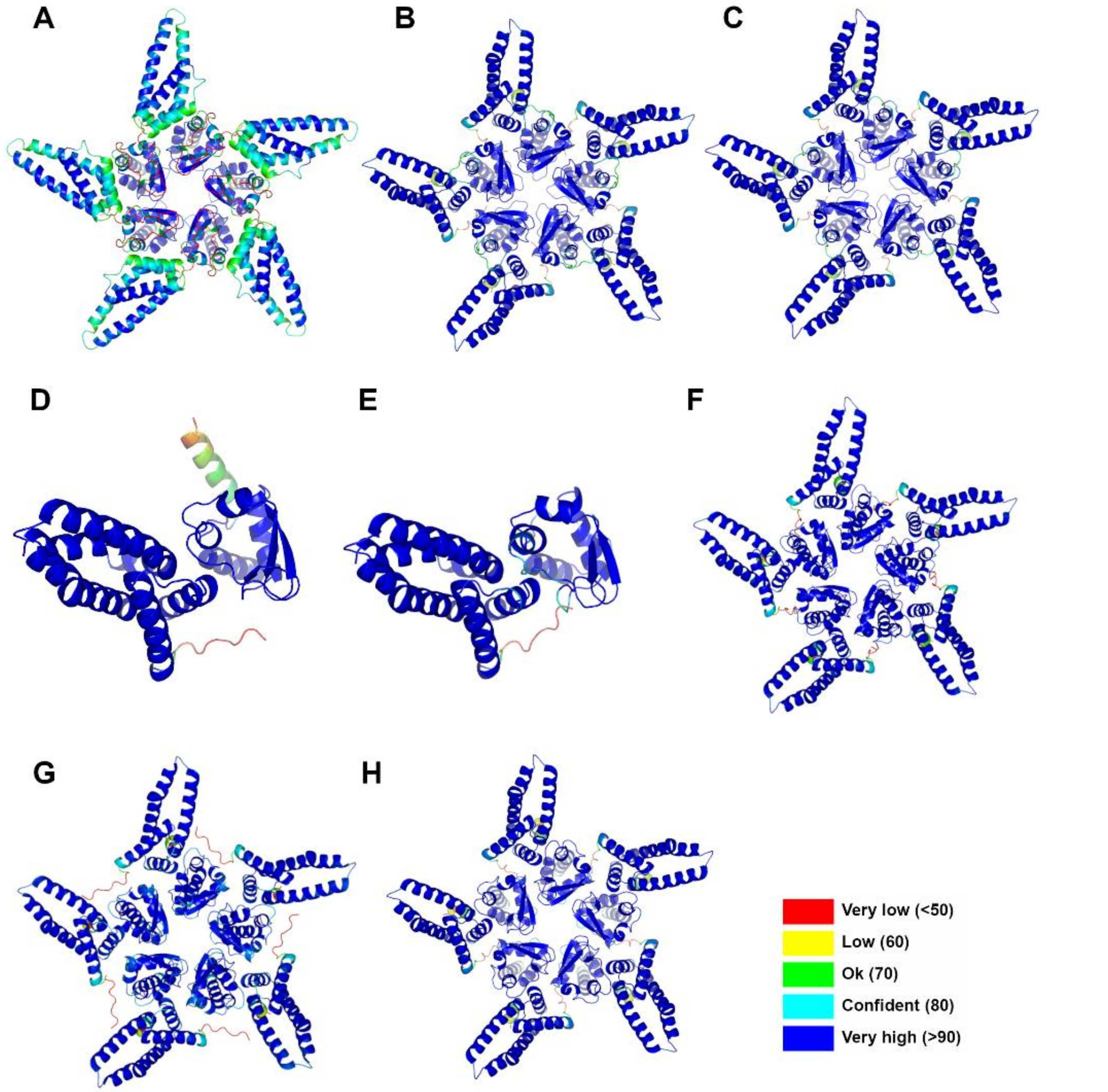

Figure S9. Ribbon representation of AF complexes of selected members of KCTDs (BTB domains) with Cullin3 (residues 17-134). (A) KCTD11 (cluster 2B); (B) KCTD2 (cluster 3); (C) KCTD17 (cluster 3); (D) KCTD18; (E) KCTD20 (cluster 5A); (F) KCTD10 (cluster 6); (G) TNFAIP1 (cluster 6); (H) SHKBP1 (cluster7). Structural models are colored following AF per-residue confidence metric (pLDDT).

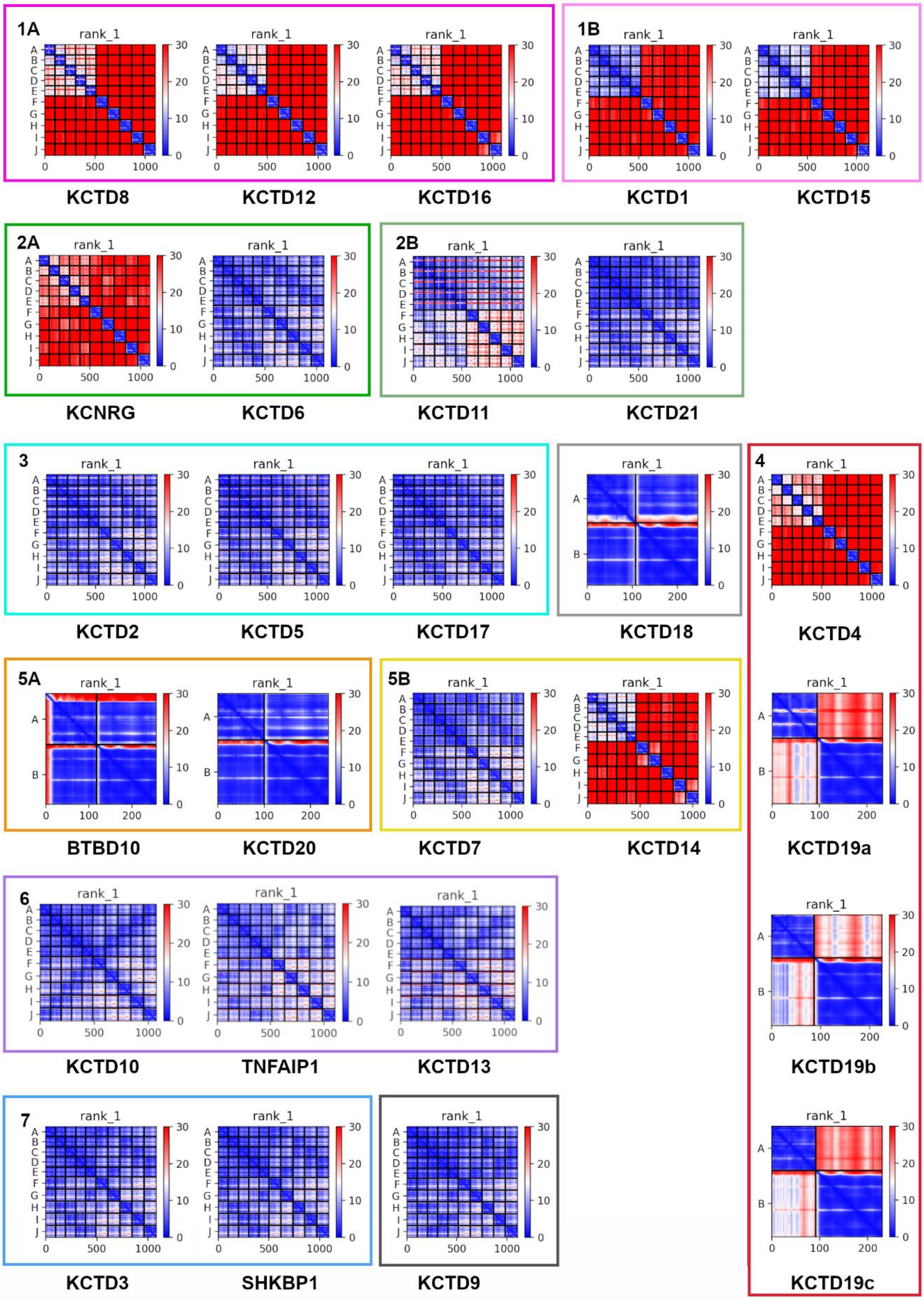

Figure S10. PAE matrices of AF complexes between KCTDs (BTB domains) and Cul3. BTB domains of KCTD proteins assume pentameric states (chains A-E) except for KCTD18, BTBD10, KCTD20, KCTD19 that assume a monomeric state (chain A). Only the best AF prediction (rank 1) is shown.

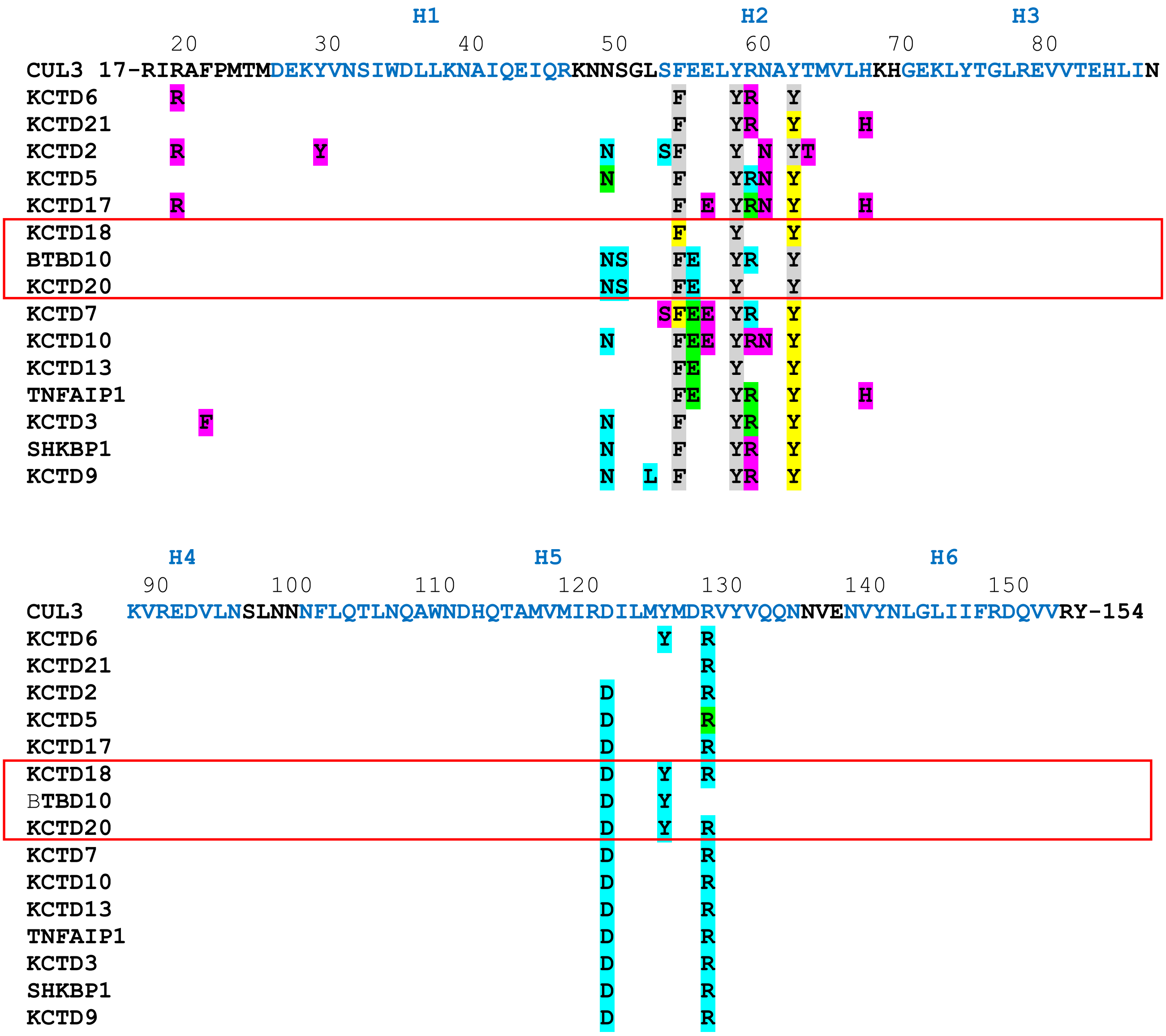

Figure S11. Residues of Cul3 involved in interactions with the BTB domain of KCTD proteins at the main (cyan for H-bonds, yellow for hydrophobic interactions, grey for both types of contacts), minor (magenta for H-bonds) and both (green for H-bonds) interfaces. Residues belonging to the six α-helices (H1-H6) are in blue.

Protein	α2-β3 region	
	**Residues**	**Sequence**
**KCTD8**	S32-R53	SPSSPRGGARRRGELPRDSRAR
**KCTD12**	Q34-R47	QQQPQELARDSKGR
**KCTD16**	S32-R48	SPKRDTANDLAKDSKGR
**KCTD1**	G33-Q44	GTEPIVLDSLKQ
**KCTD15**	G33-Q44	GTEPIVLDSLKQ
**KCNRG**	L31-G43	LDGRDQEFKMVGG
**KCTD4**	N32-H44	NGKILCPFDADGH
**KCTD14**	S33-R44	SLAKASTDAEGR

Figure S12. Sequence of the α2-β3 loop of KCTD proteins that do not form stable complexes with Cul3. Residues in 3(10)-helices and strands are highlighted in blue and red, respectively.

KCTD	1	2	3	4	5	6	7	8	9	10	11	12	13	14	15	16	17	18	19a	19b	19c	20	21	TNF	SHK	KCN	BTB10
1		33.7	33.7	41.4	31.5	53.8	37.2	35.4	30.2	33.7	43.2	40.2	29.4	31.9	82.5	39.4	34.4	34.5	33	38.5	39.1	26	50.5	28.7	37.6	37.8	27.1
2	104		47.3	36.7	79.4	36.1	35.2	32.4	47.1	33.3	39.8	38.7	35.1	40.6	35.2	37.5	72.1	35.6	34.8	32	31.9	29.9	34.7	31.1	48	37.9	26.9
3	92	91		42.2	45.6	46.7	38.6	36.0	40.2	38.6	45.7	36.6	32.3	38.2	37.8	34.7	52.8	34.9	32.2	28.6	32.8	28.6	47.8	39.8	82.5	41.4	25.0
4	99	98	90		38.1	53.3	38.9	39.4	35.4	43.6	47.4	37.9	40.9	37.5	45.4	40.0	39.0	33.3	30.4	38.9	38.5	28.4	48.4	36.2	41.8	38.5	21.6
5	105	107	90	97		40.6	34.7	32.4	47.5	33.3	37.8	33.7	36.2	41.1	31.5	37.5	76.7	39.1	33.8	30	30	29.9	35.1	31.1	41.3	33.7	23.9
6	91	97	90	90	96		45.9	43.1	38.9	42.2	60.6	44	43.3	37.4	56.7	43.8	43.2	44.8	33.7	34.8	34.8	32.4	71.9	44.9	44.6	43	27.8
7	94	91	88	95	95	98		38.8	38.9	47.3	43	34.4	46.7	48.4	38.3	45.5	38.3	41.9	29	34.8	28.7	33.3	45.2	42.7	40.9	37.6	27.5
8	99	105	100	109	108	102	103		35.3	40.6	39.6	62.4	36.6	43.1	36.7	67.3	29.6	39.4	33.3	28.2	27.3	27.2	45.2	35.3	36.9	41	26.6
9	96	102	92	96	99	95	95	102		35.6	38.1	36.8	40	43.2	31.9	38.1	47.3	41.4	28	31.9	27.4	26.9	41.7	31.7	38.5	35.7	26.6
10	92	105	88	94	105	90	93	101	101		43.6	38.4	79	42.2	33	39.6	37.9	39.8	32.2	33.7	33.7	31.5	50.5	78	37.9	32.6	30.1
11	95	98	94	97	98	99	100	101	97	94		39.6	42.6	37.4	40	38.8	40.8	38.2	34.7	37.5	37.5	29.7	63.4	43.6	42.3	41.7	28.9
12	92	93	93	95	92	100	96	101	95	99	96		36.3	42.1	41.7	68.4	38	42.2	35.3	34.4	30.9	28.8	45.8	37.4	34.4	47.3	27.4
13	102	94	99	93	94	90	92	101	100	100	94	91		42.2	29.9	37.5	36.8	39.8	27.5	33.7	33	30.1	49.5	67	33.7	31.4	32.7
14	94	96	89	80	95	99	93	102	95	90	99	95	90		32.6	47.4	39.4	42.6	27.8	30.8	28.7	26.1	41.5	37.1	39.1	41.9	35.9
15	103	91	90	97	89	90	94	98	94	97	100	96	97	95		37.6	34	34.6	34.7	34.8	33.3	27.5	51	28.9	42.9	37.9	23.7
16	94	96	95	95	96	96	66	110	97	96	103	98	96	97	93		33.3	40.2	32.7	28	44.4	29.9	46.1	36.1	36.7	45	27.6
17	90	104	89	100	103	95	94	108	91	103	98	92	106	94	97	96		37.2	25.5	34	30.2	29.9	39.4	33.3	47.4	33	26.9
18	84	87	83	84	87	87	86	94	87	83	89	90	83	68	81	92	86		37.1	20	30.2	23.5	44.8	40	34.9	36.5	29.9
19a	97	66	90	79	65	98	100	63	93	59	101	68	80	97	95	98	98	62		27.2	27.2	33.3	37.2	27.3	33.3	28.4	28.6
19b	52	50	63	90	90	92	23	39	47	89	96	64	89	65	89	75	50	35	81		34.6	29.2	36.1	28.7	33.8	28	33.3
19c	92	91	67	91	90	92	94	99	95	89	96	97	91	94	93	45	53	53	81	81		24.9	35.8	28.1	32.8	27.4	31
20	73	67	91	74	67	74	69	81	78	73	74	73	73	69	69	76	67	95	21	24	17		28	31.9	29.9	26.9	77
21	95	98	90	95	97	96	93	104	96	93	101	96	93	94	96	102	99	87	94	97	95	75		46	50	44.6	27.6
TNF	101	106	88	94	103	89	96	102	101	100	94	91	103	97	97	97	102	85	66	87	89	72	100		35.8	31.2	28.9
SHK	101	102	97	98	104	92	93	103	96	95	97	96	104	92	91	98	97	86	93	65	67	77	96	95		42.9	27.9
KCN	90	95	87	91	95	93	101	105	98	92	96	93	102	93	87	100	97	85	95	93	95	78	82	93	91		29.5
BTB10	59	67	92	74	71	90	69	94	79	73	90	73	52	64	59	76	67	97	21	27	29	100	87	76	68	78	

Figure S13. Pairwise sequence identity between all BTB domains of KCTD proteins. For each pair, the percentage of identical residues and the number of aligned residues are reported above and below the diagonal. The color code present in the vertical and orizonal bars discriminate between Cul3-binders (green) and non-binders (red). In the protein naming the KCTD acronym has been omitted. TNF, SHK, KCN, BTB10 are the abbreviations for TNFAIP1, SHKBP1, KCNRG, BTBD10, respectively. The green box reports the sequence identity of BTB domains belonging to KCTDs whose CTD domains are aligned by BLAST.

References

1. Bennett, E.J.; Rush, J.; Gygi, S.P.; Harper, J.W. Dynamics of Cullin-RING Ubiquitin Ligase Network Revealed by Systematic Quantitative Proteomics. *Cell* **2010**, *143*, 951–965, doi:10.1016/j.cell.2010.11.017.

2. Huttlin, E.L.; Bruckner, R.J.; Paulo, J.A.; Cannon, J.R.; Ting, L.; Baltier, K.; Colby, G.; Gebreab, F.; Gygi, M.P.; Parzen, H.; et al. Architecture of the Human Interactome Defines Protein Communities and Disease Networks. *Nature* **2017**, *545*, 505–509, doi:10.1038/nature22366.

3. Kouranti, I.; Abdel Khalek, W.; Mazurkiewicz, S.; Loisel-Ferreira, I.; Gautreau, A.M.; Pintard, L.; Jeunemaitre, X.; Clauser, E. Cullin 3 Exon 9 Deletion in Familial Hyperkalemic Hypertension Impairs Cullin3-Ring-E3 Ligase (CRL3) Dynamic Regulation and Cycling. *Int. J. Mol. Sci.* **2022**, *23*, 5151, doi:10.3390/ijms23095151.

4. Huttlin, E.L.; Bruckner, R.J.; Navarrete-Perea, J.; Cannon, J.R.; Baltier, K.; Gebreab, F.; Gygi, M.P.; Thornock, A.; Zarraga, G.; Tam, S.; et al. Dual Proteome-Scale Networks Reveal Cell-Specific Remodeling of the Human Interactome. *Cell* **2021**, *184*, 3022-3040.e28, doi:10.1016/j.cell.2021.04.011.

5. Luck, K.; Kim, D.-K.; Lambourne, L.; Spirohn, K.; Begg, B.E.; Bian, W.; Brignall, R.; Cafarelli, T.; Campos-Laborie, F.J.; Charloteaux, B.; et al. A Reference Map of the Human Binary Protein Interactome. *Nature* **2020**, *580*, 402–408, doi:10.1038/s41586-020-2188-x.

6. Chatrathi, H.E.; Collins, J.C.; Wolfe, L.A.; Markello, T.C.; Adams, D.R.; Gahl, W.A.; Werner, A.; Sharma, P. Novel *CUL3* Variant Causing Familial Hyperkalemic Hypertension Impairs Regulation and Function of Ubiquitin Ligase Activity. *Hypertension* **2022**, *79*, 60–75, doi:10.1161/HYPERTENSIONAHA.121.17624.

7. Pinkas, D.M.; Sanvitale, C.E.; Bufton, J.C.; Sorrell, F.J.; Solcan, N.; Chalk, R.; Doutch, J.; Bullock, A.N. Structural Complexity in the KCTD Family of Cullin3-Dependent E3 Ubiquitin Ligases. *Biochem. J.* **2017**, *474*, 3747–3761, doi:10.1042/BCJ20170527.

8. Ji, A.X.; Chu, A.; Nielsen, T.K.; Benlekbir, S.; Rubinstein, J.L.; Privé, G.G. Structural Insights into KCTD Protein Assembly and Cullin3 Recognition. *J. Mol. Biol.* **2016**, *428*, 92–107, doi:10.1016/j.jmb.2015.08.019.

9. Rolland, T.; Taşan, M.; Charloteaux, B.; Pevzner, S.J.; Zhong, Q.; Sahni, N.; Yi, S.; Lemmens, I.; Fontanillo, C.; Mosca, R.; et al. A Proteome-Scale Map of the Human Interactome Network. *Cell* **2014**, *159*, 1212–1226, doi:10.1016/j.cell.2014.10.050.

10. Kato, K.; Miya, F.; Oka, Y.; Mizuno, S.; Saitoh, S. A Novel Missense Variant in CUL3 Shows Altered Binding Ability to BTB-Adaptor Proteins Leading to Diverse Phenotypes of CUL3-Related Disorders. *J. Hum. Genet.* **2021**, *66*, 491–498, doi:10.1038/s10038-020-00868-9.

11. Young, B.D.; Sha, J.; Vashisht, A.A.; Wohlschlegel, J.A. Human Multisubunit E3 Ubiquitin Ligase Required for Heterotrimeric G-Protein β-Subunit Ubiquitination and Downstream Signaling. *J. Proteome Res.* **2021**, *20*, 4318–4330, doi:10.1021/acs.jproteome.1c00292.

12. Heride, C.; Rigden, D.J.; Bertsoulaki, E.; Cucchi, D.; De Smaele, E.; Clague, M.J.; Urbé, S. The Centrosomal Deubiquitylase USP21 Regulates Gli1 Transcriptional Activity and Stability. *J. Cell Sci.* **2016**, jcs.188516, doi:10.1242/jcs.188516.

13. Smaldone, G.; Pirone, L.; Balasco, N.; Di Gaetano, S.; Pedone, E.M.; Vitagliano, L. Cullin 3 Recognition Is Not a Universal Property among KCTD Proteins. *PLOS ONE* **2015**, *10*, e0126808, doi:10.1371/journal.pone.0126808.

14. Lange, S.; Perera, S.; Teh, P.; Chen, J. Obscurin and KCTD6 Regulate Cullin-Dependent Small Ankyrin-1 (sAnk1.5) Protein Turnover. *Mol. Biol. Cell* **2012**, *23*, 2490–2504, doi:10.1091/mbc.e12-01-0052.

15. Kim, E.-J.; Kim, S.-H.; Jin, X.; Jin, X.; Kim, H. KCTD2, an Adaptor of Cullin3 E3 Ubiquitin Ligase, Suppresses Gliomagenesis by Destabilizing c-Myc. *Cell Death Differ.* **2017**, *24*, 649–659, doi:10.1038/cdd.2016.151.

16. Balasco, N.; Pirone, L.; Smaldone, G.; Di Gaetano, S.; Esposito, L.; Pedone, E.M.; Vitagliano, L. Molecular Recognition of Cullin3 by KCTDs: Insights from Experimental and Computational Investigations. *Biochim. Biophys. Acta BBA - Proteins Proteomics* **2014**, *1844*, 1289–1298, doi:10.1016/j.bbapap.2014.04.006.

17. Bayón, Y.; Trinidad, A.G.; De La Puerta, M.L.; Del Carmen Rodríguez, M.; Bogetz, J.; Rojas, A.; De Pereda, J.M.; Rahmouni, S.; Williams, S.; Matsuzawa, S.; et al. KCTD5, a Putative Substrate Adaptor for Cullin3 Ubiquitin Ligases. *FEBS J.* **2008**, *275*, 3900–3910, doi:10.1111/j.1742-4658.2008.06537.x.

18. Cho, H.J.; Ryu, K.-J.; Baek, K.E.; Lim, J.; Kim, T.; Song, C.Y.; Yoo, J.; Lee, H.G. Cullin 3/KCTD5 Promotes the Ubiqutination of Rho Guanine Nucleotide Dissociation Inhibitor 1 and Regulates Its Stability. *J. Microbiol. Biotechnol.* **2020**, *30*, 1488–1494, doi:10.4014/jmb.2007.07033.

19. Kasahara, K.; Kawakami, Y.; Kiyono, T.; Yonemura, S.; Kawamura, Y.; Era, S.; Matsuzaki, F.; Goshima, N.; Inagaki, M. Ubiquitin-Proteasome System Controls Ciliogenesis at the Initial Step of Axoneme Extension. *Nat. Commun.* **2014**, *5*, 5081, doi:10.1038/ncomms6081.

20. Wang, Y.; Cao, X.; Liu, P.; Zeng, W.; Peng, R.; Shi, Q.; Feng, K.; Zhang, P.; Sun, H.; Wang, C.; et al. KCTD7 Mutations Impair the Trafficking of Lysosomal Enzymes through CLN5 Accumulation to Cause Neuronal Ceroid Lipofuscinoses. *Sci. Adv.* **2022**, *8*, eabm5578, doi:10.1126/sciadv.abm5578.

21. Staropoli, J.F.; Karaa, A.; Lim, E.T.; Kirby, A.; Elbalalesy, N.; Romansky, S.G.; Leydiker, K.B.; Coppel, S.H.; Barone, R.; Xin, W.; et al. A Homozygous Mutation in KCTD7 Links Neuronal Ceroid Lipofuscinosis to the Ubiquitin-Proteasome System. *Am. J. Hum. Genet.* **2012**, *91*, 202–208, doi:10.1016/j.ajhg.2012.05.023.

22. Metz, K.A.; Teng, X.; Coppens, I.; Lamb, H.M.; Wagner, B.E.; Rosenfeld, J.A.; Chen, X.; Zhang, Y.; Kim, H.J.; Meadow, M.E.; et al. *KCTD7* Deficiency Defines a Distinct Neurodegenerative Disorder with a Conserved Autophagy-lysosome Defect. *Ann. Neurol.* **2018**, *84*, 766–780, doi:10.1002/ana.25351.

23. Wang, J.; Huo, K.; Ma, L.; Tang, L.; Li, D.; Huang, X.; Yuan, Y.; Li, C.; Wang, W.; Guan, W.; et al. Toward an Understanding of the Protein Interaction Network of the Human Liver. *Mol. Syst. Biol.* **2011**, *7*, 536, doi:10.1038/msb.2011.67.

24. Olma, M.H.; Roy, M.; Le Bihan, T.; Sumara, I.; Maerki, S.; Larsen, B.; Quadroni, M.; Peter, M.; Tyers, M.; Pintard, L. An Interaction Network of the Mammalian COP9 Signalosome Identifies Dda1 as a Core Subunit of Multiple Cul4-Based E3 Ligases. *J. Cell Sci.* **2009**, *122*, 1035–1044, doi:10.1242/jcs.043539.

25. Ren, K.; Yuan, J.; Yang, M.; Gao, X.; Ding, X.; Zhou, J.; Hu, X.; Cao, J.; Deng, X.; Xiang, S.; et al. KCTD10 Is Involved in the Cardiovascular System and Notch Signaling during Early Embryonic Development. *PLoS ONE* **2014**, *9*, e112275, doi:10.1371/journal.pone.0112275.

26. Dubiel, D.; Bintig, W.; Kähne, T.; Dubiel, W.; Naumann, M. Cul3 Neddylation Is Crucial for Gradual Lipid Droplet Formation during Adipogenesis. *Biochim. Biophys. Acta BBA - Mol. Cell Res.* **2017**, *1864*, 1405–1412, doi:10.1016/j.bbamcr.2017.05.009.

27. Nagai, T.; Mukoyama, S.; Kagiwada, H.; Goshima, N.; Mizuno, K. Cullin-3-KCTD10-Mediated CEP97 Degradation Promotes Primary Cilium Formation. *J. Cell Sci.* **2018**, jcs.219527, doi:10.1242/jcs.219527.

28. Chen, Y.; Yang, Z.; Meng, M.; Zhao, Y.; Dong, N.; Yan, H.; Liu, L.; Ding, M.; Peng, H.B.; Shao, F. Cullin Mediates Degradation of RhoA through Evolutionarily Conserved BTB Adaptors to Control Actin Cytoskeleton Structure and Cell Movement. *Mol. Cell* **2009**, *35*, 841–855, doi:10.1016/j.molcel.2009.09.004.

29. Ibeawuchi, S.-R.C.; Agbor, L.N.; Quelle, F.W.; Sigmund, C.D. Hypertension-Causing Mutations in Cullin3 Protein Impair RhoA Protein Ubiquitination and Augment the Association with Substrate Adaptors. *J. Biol. Chem.* **2015**, *290*, 19208–19217, doi:10.1074/jbc.M115.645358.

30. Hu, X.; Yan, F.; Wang, F.; Yang, Z.; Xiao, L.; Li, L.; Xiang, S.; Zhou, J.; Ding, X.; Zhang, J. TNFAIP1 Interacts with KCTD10 to Promote the Degradation of KCTD10 Proteins and Inhibit the Transcriptional Activities of NF-κB and AP-1. *Mol. Biol. Rep.* **2012**, *39*, 9911–9919, doi:10.1007/s11033-012-1858-7.

31. Jiang, W.; Wang, W.; Kong, Y.; Zheng, S. Structural Basis for the Ubiquitination of G Protein Bγ Subunits by KCTD5/Cullin3 E3 Ligase. *Sci. Adv.* **2023**, *9*, eadg8369, doi:10.1126/sciadv.adg8369.

32. Esposito, L.; Balasco, N.; Vitagliano, L. Alphafold Predictions Provide Insights into the Structural Features of the Functional Oligomers of All Members of the KCTD Family. *Int. J. Mol. Sci.* **2022**, *23*, 13346, doi:10.3390/ijms232113346.

33. Esposito, L.; Balasco, N.; Smaldone, G.; Berisio, R.; Ruggiero, A.; Vitagliano, L. AlphaFold-Predicted Structures of KCTD Proteins Unravel Previously Undetected Relationships among the Members of the Family. *Biomolecules* **2021**, *11*, 1862, doi:10.3390/biom11121862.
